# Editorial: New Frontiers in Noninvasive Brain Stimulation: Cognitive, Affective and Neurobiological Effects of Transcutaneous Vagus Nerve Stimulation

**DOI:** 10.3389/fpsyg.2021.694723

**Published:** 2021-05-24

**Authors:** Mathias Weymar, Tino Zaehle

**Affiliations:** ^1^Department of Biological Psychology and Affective Science, Faculty of Human Sciences, University of Potsdam, Potsdam, Germany; ^2^Faculty of Health Sciences Brandenburg, University of Potsdam, Potsdam, Germany; ^3^Department of Neurology, Otto-von-Guericke University, Magdeburg, Germany; ^4^Center for Behavioral Brain Sciences, Magdeburg, Germany

**Keywords:** vagus nerve stimulation, tVNS, neuromodulation, cognition, affective, neurobiological

Since its first introduction two decades ago, transcutaneous vagus nerve stimulation (tVNS) has drawn tremendous attention as a promising non-invasive tool to stimulate the vagus nerve in the brain. Through the putative activation of afferent vagal projections to distinct brainstem, subcortical and cortical regions, and associated neurotransmitter systems (e.g., noradrenaline, GABA), tVNS was originally used as an alternative treatment option for epilepsy, depression, and other clinical conditions. More recently, it has been used in non-clinical populations to modulate affective and cognitive functions. However, despite the undisputed potential and accumulating evidence for tVNS-related improvements in corresponding domains, such as emotion recognition, fear extinction, cognitive control, and attention, there is also uncertainty in the field with respect to tVNS research settings, e.g. stimulation parameters, duration of stimulation (also concurrent to a certain task or not), mechanisms of action, and related biomarkers for effective stimulation, which is reflected at some point by an even number of non-replicable or mere subtle findings.

The aim of this Frontiers Research Topic on “Non-invasive Brain Stimulation: Cognitive, Affective and Neurobiological Effects of Transcutaneous Vagus Nerve Stimulation” is to present recent advances in the application of tVNS as a useful neuromodulation tool for the systematic study of cognition and emotion as well as its underlying neurobiological processes, to address new trends and emerging neuromodulation technologies, and ultimately foster fruitful discussion and interaction among researchers.

The articles in this Frontiers Research Topic perfectly reflect the current status of the field by encompassing articles that span from the novel but also mixed findings in the newly emerging fields of tVNS (mainly referring to auricular stimulation) application in basic and clinical research to reviews that highlight important anatomical considerations, strategies, and recommendations to overcome current challenges when applying tVNS in future research.

The submissions from basic research include behavioral findings on the impact of tVNS on episodic memory. Mertens et al. showed that auricular tVNS, compared to sham and control stimulation, had no improving effect on immediate free recall and recognition memory for attended verbal material when applied directly after encoding. Giraudier et al. partly replicated this finding; however, they demonstrated that tVNS during a lexical decision task can improve delayed recognition memory performance for emotional and neutral words when memory quality was taken into account (i.e., tVNS effects on high confidence memory reflecting recollection). Contrary to other studies, in this article, tVNS did not affect a putative indirect marker for central noradrenergic activity (salivary alpha amylase). Two other studies emphasized the role of tVNS in food processing, hedonic assessment, and ingestive behavior (Obst et al., Öztürk et al.). Pre-task tVNS in food deprived individuals showed no effect on the processing of food as revealed by event-related brain potentials (ERP), compared to sham, but found overall ERP differences in components related to visual attention and conflict processing (Obst et al.). Preliminary evidence by Öztürk et al. however, emphasized that concurrent tVNS stimulation increased food related hedonic responses, such as liking of low-fat food during a food sample task, also pointing toward a plausible triggering of dopaminergic reward circuits in the brain by tVNS (although spontaneous eye blink rate as a potential biomarker of dopamine functioning was not modulated by tVNS). Food consumption, however, was not influenced by vagal stimulation in both studies (Obst et al.; Öztürk et al.). Submissions from application in clinical settings further suggest that motivated behavior (such as oral intake) can be improved by tVNS by facilitating oromotor learning over a period of 2 weeks in premature or brain injured infants with oral feeding difficulties when vagal stimulation was paired with bottle-feeding (Badran et al.). Further evidence suggests that tVNS may also be effective as adjunct treatment of symptoms of acute opioid withdrawal, as shown in a study by Jenkins et al., in which auricular nerve stimulation (which also includes vagal branch) resulted in alleviation of withdrawal symptoms and duration of opioid replacement therapy in infants suffering from opioid toxification, which may be explained by the involvement of dopaminergic circuits as an extended vagal system (c.f., Öztürk et al.; Badran et al.). Finally, Ylikoski et al. present outcome data from a treatment program showing that tVNS may be a useful long-term therapeutic adjunct reducing distress in patients suffering from tinnitus. It is also assumed by the authors that tVNS may exert its anti-stress effects via a (parasympathetic) efferent pathway based on treatment baseline data showing short-term improvements in heart rate variability by tVNS (see also Badran et al. for reporting increased heart rate deceleration). It should be noted though that all clinical studies in this Research Topic did not include a control or sham condition, which needs to be considered when interpreting the potential impact of tVNS in these studies.

The current Research Topic on tVNS also includes critical reviews and a perspective paper. Paciorek and Skora emphasize an important link between vagus nerve stimulation and interoception, which also guides various cognitive and affective functions at different levels of processing, encouraging elaborate tVNS research in the future. Cakmak points to important anatomical pathways that modulate central vagal and non-vagal structures depending on the activation of specific auricular zones, helping researchers in the field to improve study designs (in terms of optimal electrode location) and to sharpen the interpretability of results (with respect to the mechanism of action). Finally, as an effort to increase knowledge and reproducibility in the field this Research Topic includes a much needed tVNS consensus paper (Farmer et al.) by Julian Koenig—with the cooperation of a large group of tVNS researchers—on guidelines and recommendations for future tVNS studies in basic research and clinical practice based on the existing tVNS literature.

In conclusion, the Research Topic shows new exciting fields of application for tVNS in basic and clinical settings. However, as reflected by the original research papers and reviews, it is clear that more research is needed to identify the exact mechanism of action including reliable biomarkers, and optimal stimulation parameters and contexts for tVNS to meet the high expectations as effective neuromodulation and treatment tool.

## Author Contributions

MW and TZ drafted and revised the manuscript. Both authors contributed to the article and approved the submitted version.

## Conflict of Interest

The authors declare that the research was conducted in the absence of any commercial or financial relationships that could be construed as a potential conflict of interest.

